# Crosstalk between cancer-associated fibroblasts and regulated cell death in tumors: insights into apoptosis, autophagy, ferroptosis, and pyroptosis

**DOI:** 10.1038/s41420-024-01958-9

**Published:** 2024-04-22

**Authors:** Cong Chen, Jian Liu, Xia Lin, Aizhai Xiang, Qianwei Ye, Jufeng Guo, Tao Rui, Jian Xu, Shufang Hu

**Affiliations:** 1 Department of Breast Surgery, Affiliated Hangzhou First People’s Hospital, School of Medicine, Westlake University, Hangzhou, China; 2 Department of Central Laboratory, Affiliated Hangzhou First People’s Hospital, School of Medicine, Westlake University, Hangzhou, China

**Keywords:** Cell death, Cancer microenvironment

## Abstract

Cancer-associated fibroblasts (CAFs), the main stromal component of the tumor microenvironment (TME), play multifaceted roles in cancer progression through paracrine signaling, exosome transfer, and cell interactions. Attractively, recent evidence indicates that CAFs can modulate various forms of regulated cell death (RCD) in adjacent tumor cells, thus involving cancer proliferation, therapy resistance, and immune exclusion. Here, we present a brief introduction to CAFs and basic knowledge of RCD, including apoptosis, autophagy, ferroptosis, and pyroptosis. In addition, we further summarize the different types of RCD in tumors that are mediated by CAFs, as well as the effects of these modes of RCD on CAFs. This review will deepen our understanding of the interactions between CAFs and RCD and might offer novel therapeutic avenues for future cancer treatments.

## Facts

- The origins, biomarkers, and functions of cancer-associated fibroblasts (CAFs) are highly heterogeneous, and diverse CAF subpopulations may exert either tumor-promoting or tumor-restrictive effects.
- There are a number of types of regulated cell death (RCD) in tumors, and dying tumor cells play various physiological and pathological roles in regulating the tumor microenvironment (TME).
- CAFs can modulate multiple forms of RCD in adjacent tumor cells, which has significant implications for cancer proliferation, therapeutic resistance, and immune exclusion.
- Tumor cells undergoing different types of RCD can also regulate CAFs, thereby developing a feedback system.

## Open questions

- How can we identify the specific subtype of CAFs that promote RCD in tumor cells, as well as the specific subtypes of CAFs that inhibit RCD?
- Tumor cells experience several forms of RCD when treated with clinical drugs, so what are the specific effects of these diverse forms of RCD on CAFs?
- How to develop more effective therapeutic strategies that combine traditional anti-tumor medicines with CAF-targeting drugs?

## Introduction

The tumor microenvironment (TME), as a complex and dynamic ecosystem composed of a set of cellular and non-cellular molecular components, plays a crucial role in tumor initiation, progression, and response to therapy [1–6]. It is well documented that cancer-associated fibroblasts (CAFs) were identified as the main stromal component in the TME and have been extensively explored in the past few years [7–9]. In many cancer types, CAFs exhibit distinct characteristics and behaviors that facilitate the development of tumors [10–12]. They can interact with tumor cells or other stromal cells via cell-cell contact, secrete numerous regulatory mediators, and remodel the structure of the extracellular matrix (ECM) [13, 14]. Clinically, CAFs are also associated with the clinicopathological characteristics of tumors and serve as potential biomarkers for diagnosis, treatment, and prognostic prediction [15–17]. Hence, the investigation of CAFs represents an active research for developing novel therapeutic strategies for cancer treatment.

Regulated cell death (RCD), also known as programmed cell death, refers to the autonomous and orderly death of cells controlled by genes that eliminate unnecessary cells and maintain the stability of organismal homeostasis [18]. In the context of cancer, RCD dysfunction is a hallmark feature that contributes to the development and progression of tumor cells [19–22]. To date, ongoing research has focused on understanding the mechanisms of RCD and developing promising anticancer strategies involved in the areas of RCD [23–26]. As the field has advanced, several forms of RCD, including apoptosis, autophagy, ferroptosis, pyroptosis, and necroptosis, have been identified and shown to play crucial roles in modulating the TME, making the study of RCD an attractive topic in cancer research [27–29].

Recently, accumulating evidence has shown that activated CAFs can regulate RCD in adjacent tumor cells, which has significant implications for cancer proliferation, therapeutic resistance, and immune exclusion. Therefore, understanding the crosstalk between CAFs and tumor cells, especially in the context of RCD, may provide new insights for improving cancer treatment outcomes. In this review, we first present a current overview of the complexity and interactions between CAFs and RCD, involving the effects of CAFs on RCD in tumor cells, as well as the impact of dying cells on CAFs. Nonetheless, for simplicity, we will only elaborate on the RCD associated with apoptosis, autophagy, ferroptosis, and pyroptosis in this study.

## The properties of CAFs

First recognized by Virchow in 1858, Fibroblasts were identified as spindle-shaped cells in connective tissue that can secrete collagen [30]. In normal tissues, fibroblasts are mesenchyme-derived quiescent cells that are generally embedded within the fibrillar ECM. They can undergo transient activation in a context-dependent manner during processes associated with wound healing, tissue inflammation, and organ fibrosis, which plays an important role in maintaining tissue homeostasis [31, 32]. Nevertheless, repetitive damage or constant inflammation and stress could lead to continuous activation of fibroblasts [33, 34]. In cancers, this subpopulation of hyperactivated fibroblasts can be termed as CAFs [35].

**Fig. 1:**
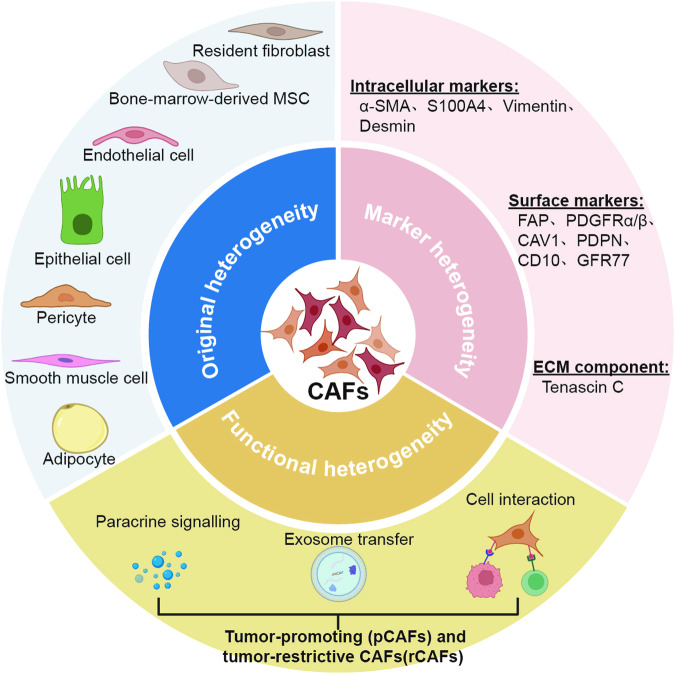
Heterogeneity of CAFs on the basis of their origins, biomarkers and functions. The potential cellular origins of CAFs include quiescent tissue-resident fibroblasts, bone-marrow-derived fibrocytes and mesenchymal stem cells (MSCs), endothelial cells, epithelial cells, and other cells (pericytes, smooth muscle cells, and adipocytes) derived through transdifferentiation. Activated CAFs can be identified by diverse biomarkers, including various intracellular markers, surface markers, and ECM proteins. It has also been reported that CAFs exhibit different biological functions in cancer progression through paracrine signaling, exosome transfer, and cell interactions.

According to the relevant literature, the potential cellular origins of CAFs include quiescent tissue-resident fibroblasts, bone-marrow-derived fibrocytes and mesenchymal stem cells (MSCs), endothelial cells, epithelial cells, and other cells (pericytes, smooth muscle cells, and adipocytes) derived through transdifferentiation [8, 36]. However, the diverse origins of CAFs make it considerably difficult to precisely categorize a specific subgroup of CAFs. In addition to the original heterogeneity, emerging evidence illustrates that CAFs can be assessed by a combination of different biological markers. Previous studies have suggested that a number of different biomarkers, such as α-SMA, Vimentin, S100A4, FAP, PDGFRα/β, Caveolin-1 and podoplanin (PDPN), have the potential to distinguish CAFs from normal fibroblasts (NFs) [37]. Notably, these so-called biomarkers also exhibit variability across disparate tumor tissues and manifest dynamic alterations as tumors progress [38–40], making targeted intervention in CAFs more challenging. Therefore, precise classification strategies and efficient identification of biomarkers in CAFs require further in-depth investigation (Fig. 1).

It has long been recognized that CAFs exhibit many malignant properties when compared with NFs. Numerous publications have shown that pro-tumorigenic factors secreted by CAFs can be transferred into tumor cells through the paracrine signaling [41, 42] and the exosome-mediated pathway [43, 44], which facilitates cancer cell stemness, proliferation, and migration. As confirmed by in vitro and in vivo experiments, CAFs also stimulate tumor angiogenesis by secreting pro-angiogenic factors, such as VEGFA, CXCL12, FGF2, and PDGFC [45]. Additionally, extensive research has revealed intricate signaling pathways involved in CAF-mediated ECM/TME remodeling as well as their contributions to therapeutic resistance and immunosuppression [46–48]. Despite the fact that CAFs play potent pro-tumorigenic roles, some CAF subsets have been reported to have tumor-suppressive properties. For example, Ozdemir et al. revealed that the depletion of CAFs in pancreatic cancer results in the advancement of tumor cells and the suppression of the TME (a decrease in the Teff/Treg ratio and an increase in Foxp3 and Ctla4 expression) and proposed the need for caution in targeting CAFs in this kind of cancer [49]. Therefore, the impact of CAFs varies and can, in certain cases, exhibit contrasting effects on the process of carcinogenesis (Fig. 1), and a comprehensive understanding of the tumor-promoting and tumor-suppressing activities of CAF subtypes may facilitate the development of novel diagnostic and therapeutic approaches.

## Apoptosis, autophagy, ferroptosis and pyroptosis in brief

As the field of cell death research has advanced, the Nomenclature Committee on Cell Death (NCCD) proposed an updated definition and classification of cell death in 2018 from morphological, biochemical, and functional perspectives [18]. In contrast to accidental cell death (ACD), RCD is characterized by controlled signaling pathways and serves a crucial function in homeostasis maintenance and disease progression. According to its different physiological and pathological mechanisms, RCD can be further subclassified into apoptotic and non-apoptotic subcategories, such as autophagy-dependent cell death, ferroptosis, pyroptosis, and necroptosis [24, 27].

Currently, apoptosis is the most intensively investigated RCD and it is generally characterized by distinct morphological characteristics, including cell shrinkage, chromatin condensation, nuclear fragmentation, membrane blebbing, and the formation of apoptotic bodies [50]. Apoptosis mainly occurs through two canonical pathways: the intrinsic (mitochondrial) pathway and the extrinsic (cell death receptor) pathway. The intrinsic pathway is initiated by intracellular signals, such as DNA damage, cellular stress, and loss of survival signals, which results in the release of pro-apoptotic proteins (e.g., cytochrome c and SMAC/DIABLO) from the mitochondria into the cytoplasm and subsequently activates the caspase 9 protein [51]. The extrinsic pathway provides a rapid response to external signals, specifically tumor necrosis factor-alpha (TNF-α), Fas ligand (FasL), and TNF-related apoptosis-inducing ligand (TRAIL), through binding to death receptors on the cell surface. Upon initiation, the death-inducing signaling complex (DISC) forms, leading to the activation of caspase-8 and subsequent initiation of the caspase cascade [23].

Many studies have demonstrated that autophagy plays a fundamental role in cellular, tissue, and organismal homeostasis [52]. Based on the way intracellular materials are transported to lysosomes and the intricacies of the autophagic process, autophagy can be categorized into macroautophagy, microautophagy, and chaperone-mediated autophagy [53]. Notably, within this classification, macroautophagy stands out as the predominant form. Autophagy initiation is mediated by the unc-51-like kinase (ULK) complex. Following the ULK complex, the autophagy-specific class III phosphoinositide 3-kinase (PI3K) complex forms, catalyzing the production of phosphatidylinositol-3-phosphate (PI3P) on autophagic membranes and generating an isolated pre-autophagosomal structure called the phagosome [54]. Subsequently, the phagosome extends and seals, transforming into the autophagosome, a double-membraned vesicle encapsulating the sequestered cargo. The autophagosome then fuses with a lysosome, creating an autolysosome. Within the autolysosome, cellular component cargoes are degraded, and nutrients are recycled [55]. Thus, not surprisingly, autophagy is a dynamic and highly regulated process responsible for the degradation and recycling of cellular components via a lysosome-mediated pathway [56], and dysregulation of the autophagic network is believed to be associated with cancer development and progression [57].

Ferroptosis is distinct from the other types of RCD mentioned above. Following its identification in 2012, it has become an extensively investigated subject in the field of cancer research [26, 58, 59]. Morphologically, cells undergoing ferroptosis display distinctive mitochondrial characteristics, including mitochondrial shrinkage, increased membrane density, and decreased or absent mitochondrial cristae [60]. Mechanistically, the central biochemical and metabolic event in ferroptosis is oxidative damage to cellular membranes resulting from the abnormal accumulation of lethal lipid peroxidation products within cells [61]. The classic antioxidant network, the glutathione peroxidase 4 (GPX4)-reduced glutathione (GSH) system (the GPX4-GSH system), mediates cellular cysteine uptake and GSH synthesis. Under the catalytic influence of GPX4, GSH efficiently reduces accumulated phospholipid hydroperoxides (PLOOHs) and prevents ferroptosis [62]. Nevertheless, research has revealed that certain cancer cell lines maintain resistance to ferroptosis even after GPX4 inactivation, indicating the existence of additional ferroptosis defence mechanisms [26], such as the ferroptosis suppressor protein-1 (FSP1)-ubiquinone (CoQ10) system (the FSP1-CoQ10 system) [63, 64], the dihydroorotate dehydrogenase (DHODH)-ubiquinol (CoQH2) system (the DHODH-CoQH2 system) [65], and the GTP cyclohydroxylase-1 (GCH1)-tetrahydrobiopterin (BH4) system (the GCH1-BH4 system) [66].

**Fig. 2:**
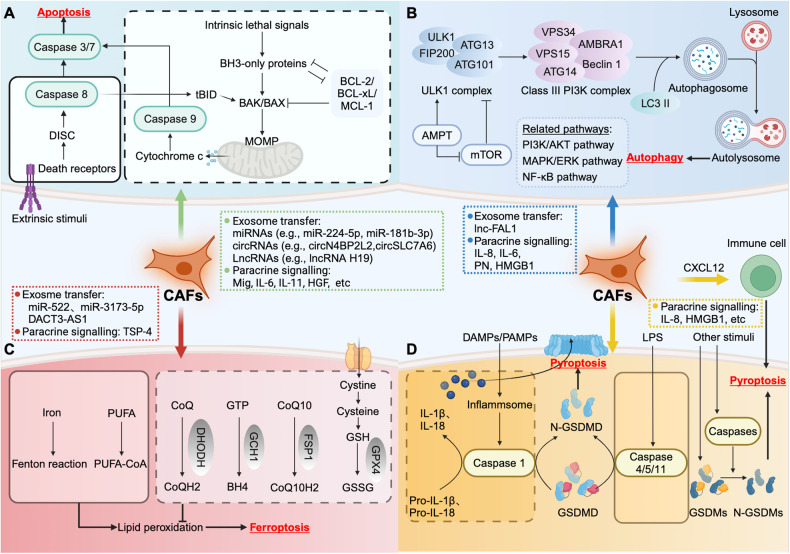
Core molecular mechanisms of different types of RCD and the potential CAF-derived molecules that regulate RCD. **A** The extrinsic pathway (left) is triggered by the activation of death receptors, such as TNFR1, Fas, and the TRAIL receptors DR4 and DR5. This leads to the formation of DISCs and the activation of caspase 8. The intrinsic pathway (right) is initiated by intracellular signals, such as DNA damage, cellular stress, and loss of survival signals, resulting in mitochondrial outer membrane permeabilization (MOMP) and subsequent release of cytochrome c from mitochondria. This triggers the activation of caspase 9. Activated caspase 8 and caspase 9 cleave downstream caspase 3 and caspase 7, which mediate the execution of apoptosis. **B** Autophagy initiation is mediated by the ULK complex, which eventually activates the class III PI3K complex. This complex produces PI3P on autophagic membranes to recruit the autophagy conjugation machinery, which is subsequently transformed into the autophagosome. The autophagosome then fuses with lysosomes to form autolysosomes. **C** The central biochemical and metabolic event in ferroptosis is oxidative damage to cellular membranes resulting from the abnormal accumulation of lethal lipid peroxidation products within cells. This effect is mainly caused by an imbalance between ferroptosis-driving (left) and ferroptosis-defense (right) mechanisms. The classic antioxidant network includes the GPX4-GSH system, the FSP1-CoQ10 system, the DHODH-CoQH2 system, and the GCH1-BH4 system. **D** Pyroptosis is mediated through two main mechanisms: the canonical (left) and noncanonical pathways (middle). The canonical pyroptosis pathway responds to cell DAMPs and PAMPs, leading to inflammasome formation. Inflammasomes then recruit proteins to activate caspase-1, which subsequently cleaves GSDMD and promotes IL-1β/IL-18 maturation. In the noncanonical pathway, caspases-4/5/11 are activated by cytosolic LPS, triggering pyroptosis by cleaving GSDMD. CAFs have been shown to modulate different types of RCD, including apoptosis, autophagy, ferroptosis, and pyroptosis, mainly through exosomal secretion and paracrine signaling pathways.

Research on pyroptosis, a newly described category of RCD that is dependent on Gasdermin proteins (GSDMs) [67, 68], has made further progress in recent years. As a form of lytic and pro-inflammatory type of programmed cell death, pyroptosis is mediated through two main mechanisms: canonical and noncanonical pathways. The canonical pyroptosis pathway responds to cell recognition of pathogen-associated molecular patterns (PAMPs) and damage-associated molecular patterns (DAMPs) through cytosolic pattern recognition receptors (PRRs), leading to inflammasome formation [69]. Inflammasomes then recruit proteins to activate caspase-1. Once activated, caspase-1 initiates pyroptosis by cleaving GSDMD and releasing mature interleukin-1β/interleukin-18 (IL-1β/IL-18) [70, 71]. In the noncanonical pathway, bacterial lipopolysaccharide (LPS) can directly bind and activate caspase-4/5/11 and further cleave GSDMD into N-GSDMD, thereby initiating the pyroptosis program [72, 73]. Furthermore, accumulating data suggest that other members of GSDMs are also involved in pyroptosis [74–77], and the expanding understanding of the diverse activities of GSDMs has made the study of pyroptosis an attractive topic for cancer research. The main pathways involved in apoptosis, autophagy, ferroptosis, and pyroptosis are summarized and presented in Fig. 2.

## Effects of CAFs on tumor apoptosis

Apoptosis, the most renowned type of RCD, is a highly regulated and controlled process that involves a series of molecular processes, leading to the orderly and controlled deconstruction of cells [25]. In certain cases, CAFs can modulate apoptosis in neighboring tumor cells through secreted factors and cell interactions within the tumor microenvironment (Fig. 2).

Dysregulation of apoptosis is one of the classical hallmarks of the maintenance and regulation of cancer development [78, 79]. Currently, a substantial body of research has illuminated the role of CAFs in the initiation and progression of cancers through the regulation of apoptosis. In colorectal cancer (CRC), the exosomes transport miRNAs (miR-224-5p [80], miR-181b-3p [81], and miR-135b-5p [82]) and circRNAs (circN4BP2L2 [83] and circSLC7A6 [84]) from CAFs into cancer cells to act as inhibitors of apoptosis and promote the pathogenesis of CRC. Moreover, the secretion of IL-6 by CAFs leads to the upregulation of BCL-XL and MCL-1 in CRC cells via the IL-6/STAT3 signaling pathway, which simultaneously promotes apoptosis resistance [85]; however, the dynamic interactions between pro-apoptotic and anti-apoptotic BCL‑2 protein families (BCL-2, BCL-XL, BCL-w, MCL-1 and BCL-2A1) balance the commitment of cells to apoptosis [86, 87]. In estrogen receptor-positive breast cancer, it has also been verified that CAFs promote MCL-1 expression and apoptosis resistance in an IL-6-dependent manner [88]. Additionally, Bian et al. derived CAFs from oral tongue squamous carcinoma tissue. Their investigation revealed that CAFs upregulated the expression of BCL-2 and inhibited heat-induced apoptosis through the paracrine secretion of Mig [89]. Moreover, it has been verified that lung squamous cell carcinoma (LUSC)-derived CAFs can enhance cell proliferation and suppress apoptosis both in vitro and in vivo. This effect is correlated with the upregulation of COL10A1 expression in CAFs. Nevertheless, the authors did not explain the mechanism by which COL10A1 is delivered into LUSC cells [90]. Notably, despite the identification of pro-malignant molecules within CAFs in numerous studies, research on the regulatory mechanisms controlling the secretion of relevant molecules within CAFs remains comparatively limited.

On the other hand, many cancer treatments, such as chemotherapy and radiation therapy, exert their effects by inducing apoptosis in cancer cells. Therefore, it is understandable that CAFs could also contribute to drug resistance by regulating the apoptosis of tumor cells. Notably, Neophytou et al. addressed the significance of CAFs in impeding apoptosis in multidrug-resistant cancers [91]. For example, in lung cancer, the present results supported that CAFs suppress the apoptosis program and enhance chemoresistance via the secretion of soluble factors and exosomes [92–96]. Similarly, studies in other cancers have also shown that CAFs promote radiation resistance in tumor cells by inhibiting the level of apoptosis [97]. Taken together, the above data indicate that CAFs can elevate the threshold for apoptosis in tumor cells, contributing specifically to the advancement of tumors and the development of treatment resistance.

Although CAFs exhibit oncogenic properties through the suppression of apoptosis in tumor cells, there is evidence suggesting that CAFs can also stimulate apoptosis in tumor cells. For instance, Itoh et al. conducted a co-culture experiment involving gastric cancer cells and CAFs. Their findings identified a significant induction of apoptosis in cancer cells upon direct interaction with CAFs, with the process controlled by the DR4-caspase-8 signaling pathway [98]. Interestingly, apoptotic cancer cells release apoptotic vesicles, which in turn promote CAF-led cancer invasion [98]. Overall, CAFs display a dual capacity in the modulation of tumor apoptosis, engaging in both anti-apoptotic and pro-apoptotic actions through secreted factors or direct cellular interactions.

## Effects of CAFs on tumor autophagy

Autophagy is a regulated mechanism that delivers dysfunctional or unnecessary cellular cargoes to lysosomes for degradation and recycling [53]. However, the process and roles of autophagy in tumor development and progression appear to be complex [55, 99]. Recent studies have addressed not only the intrinsic functions of autophagy in tumor cells but also the involvement of autophagy in the TME, including immune cells and stromal cells [100–102]. Although several publications have documented that autophagy occurs in CAFs [103–105], herein, we aimed to summarize the impacts exerted by CAFs on the regulation of autophagy signaling in tumor cells (Fig. 2).

Dysfunctional regulation of autophagy in ovarian cancer mediated by CAFs has been discussed previously [106]. In recent years, Thongchot et al. have identified IL-8 as a cytokine released by CAFs that drives ovarian cancer cell metastasis and is mechanistically linked to the downregulation of autophagy [107]. Consistently, other studies revealed that the cytokine IL-6 isolated from CAFs promoted cholangiocarcinoma cell migration, and this effect was also associated with the inhibition of autophagy in cancer cells [108–110]. Moreover, periostin (PN) is a secreted extracellular matrix protein that is commonly expressed in CAFs in several cancers [111, 112]. In colorectal cancer, CAF-derived PN was confirmed to promote the migration of cancer cells. Specifically, CAF-derived PN bound to the predominant ITGα5β1 or ITGα6β4 receptors on colorectal cancer cells, thus initiating the AKT-dependent signaling cascade and further attenuating autophagy [113]. These findings suggest that CAF-derived cytokines/chemokines confer malignant phenotypes on tumor cells via autophagy suppression and that CAFs may be potential therapeutic targets for treating or blocking cancer metastasis.

In conjunction with investigating the roles of CAF-mediated autophagy in tumor metastasis, numerous studies have focused on delineating the significance of CAF-mediated autophagy in the context of tumor drug resistance. In colorectal cancer, Zhu et al. reported that the long non-coding RNA FAL1 (lnc-FAL1) was mainly derived from exosomes released by CAFs. However, the overexpression of lnc-FAL1 in cancer cells was further observed to hinder autophagy by promoting the ubiquitination and degradation of Beclin1, thereby contributing to oxaliplatin resistance [114]. Indeed, the impact of autophagy on tumor drug resistance is inherently dualistic [115, 116]. Concurrently, CAFs also have dual effects on tumor drug resistance, not only through the inhibition of autophagy but also by enhancing autophagic processes. Liu et al. revealed that in breast cancer, high mobility group box 1 (HMGB1) functions as an extracellular signaling molecule and is secreted by CAFs via the GPR30/PI3K/AKT signaling pathway. Meanwhile, CAF-derived HMGB1 was further confirmed to stimulate autophagy through MEK/ERK signaling in ERα-positive breast cancer cells, thus promoting tamoxifen resistance in cancer cells [117]. Similarly, an additional study revealed increased HMGB1 expression in breast cancer cells after coculture with CAFs, wherein the heightened HMGB1 levels increased resistance to doxorubicin in cancer cells through the potentiation of autophagy [118]. In addition, Liao et al. first reported that CAFs contribute to the development of cisplatin resistance in tongue cancer, which is also associated with the activation of autophagy mechanisms [119]. In summary, the effect of CAFs on tumor resistance through the autophagy pathway is multifaceted, and unraveling the precise molecular pathways involved in this process has potential for developing targeted therapies by focusing on CAFs.

Intriguingly, Mukhopadhyay et al. proposed a novel function of CAFs in pancreatic ductal adenocarcinoma (PDAC) [120]. It has been shown that blocking autophagy in PDAC cells is a powerful approach for hindering the development of tumors. Mechanistically, they verified that autophagy suppression attenuates the formation of a labile iron pool (LIP) (Fe2+) and impedes the synthesis of iron-sulfur clusters [120]. Consequently, this cascade leads to a reduction in the succinate dehydrogenase complex iron sulfur subunit B (SDHB), resulting in compromised mitochondrial function [120]. However, when cocultured with autophagy-inhibited PDAC cells in vitro, a notable upregulation of the iron efflux protein ferroportin (FPN) was observed in CAFs, facilitating the compensation of the LIP in autophagy-inhibited PDAC cells [120]. Thus, their proposal suggests an intricate interaction in which CAFs serve as a substitute for autophagy in PDAC [120, 121].

## Effects of CAFs on tumor ferroptosis

Ferroptosis, which is characterized by intracellular iron accumulation and lipid peroxidation, is a newly described form of regulated cell death that was first defined in 2012 [58]. In recent years, ferroptosis has attracted a great deal of attention in cancer research communities, and it exhibits a promising prospect for cancer treatment [26].

Studies have shown that CAFs can support cancer proliferation and therapeutic resistance by inhibiting ferroptosis. A close association between arachidonate lipoxygenase 15 (ALOX15) and lipid peroxidation has been reported in various types of cancers, and was reported as a potential promoter of ferroptosis [122, 123]. In gastric cancer, researchers revealed that CAFs inhibit ferroptosis and promote tumor growth by secreting exosomal miR-522, which targets ALOX15. Moreover, cisplatin and paclitaxel can promote miR-522 secretion from CAFs by activating the USP7/hnRNPA1 pathway [123]. Besides, long-chain fatty acid-CoA ligase 4 (ACSL4) is involved in the activation of long-chain fatty acid metabolism and is reported to enhance the sensitivity of cancer cells to ferroptosis by promoting the accumulation of lipid peroxidation products [124]. Qi et al. demonstrated that miR-3173-5p derived from CAF exosomes sponged ACSL4 and inhibited ferroptosis, which induced gemcitabine resistance in pancreatic cancer [125]. Of note, in addition to secreting exosomes, CAFs also play a regulatory role in tumor cell ferroptosis through the secretion of small molecular proteins. Previous research has revealed that CAFs activate HSF1 in tumor cells by secreting thrombospondin-4 (TSP-4) [126]. In another study, CAFs were shown to confer resistance to ferroptosis in glioblastoma cell lines through HSF1 activation. Mechanistically, HSF1 transcriptionally enhances DLEU1 expression, which leads to a decrease in ATF3 and an increase in SLC7A11 [127]. Hence, eliminating CAFs from the TME will result in increased ferroptosis and improved sensitivity to cancer treatment.

Interestingly, CAFs can also deliver tumor suppressor factors and stimulate ferroptosis in tumor cells. Qu et al. reported a novel lncRNA Disheveled Binding Antagonist of beta Catenin3 antisense1 (DACT3-AS1), and showed that CAF-derived exosomal DACT3-AS1 could alleviate gastric cancer cell proliferation, migration, and invasion. Furthermore, CAF-derived exosomal DACT3-AS1 was confirmed to downregulate the expression of xCT and GPX4 in oxaliplatin-treated cells and increase oxaliplatin sensitivity by activating ferroptosis both in vitro and in vivo [128]. In summary, CAFs have great potential as cancer therapeutic targets by mediating ferroptosis, and we suspect that the current challenge is to elucidate the intricate mechanisms underlying the dual regulation of ferroptosis by different signaling factors in CAFs (CAF-derived molecules that regulate ferroptosis are summarized in Fig. 2).

## Effects of CAFs on tumor pyroptosis

Pyroptosis, an inflammatory form of RCD mediated by GSDMs, is associated with tumor-associated inflammation. Intriguingly, previous research has indicated the active involvement of CAFs in contributing to tumor inflammation [129]. In breast cancer, DAMPs can trigger the activation of the NLRP3 inflammasome and induce pyroptosis in CAFs through the NLRP3/caspase-1/GSDMD pathway. The resulting release of IL-1β further promotes tumor growth and metastasis [130].

Nevertheless, there is still limited research on the specific processes and mechanisms by which CAFs directly regulate pyroptosis in tumor cells. Limited data from public databases indicate a positive correlation between CAF infiltration and the expression of GSDMD and GSDME in cancer cells [131, 132], suggesting the potential for CAF-induced pyroptosis in cancer cells. In addition, Hou et al. identified a non-immune checkpoint function of PD-L1 and demonstrated that PD-L1, cooperated with p-Stat3, could transcriptionally upregulate the expression of GSDMC, leading to the transition of cancer cells from apoptosis to pyroptosis [75]. Meanwhile, several published studies have shown that CAFs can upregulate PD-L1 expression in cancer cells by transporting some cytokines and vesicles [133–136]. Therefore, we hypothesize that CAFs can modulate the process of pyroptosis by indirectly regulating pyroptotic signaling pathways. Similarly, it has been verified that CAFs exhibit elevated levels of pro-inflammatory factors and can secrete signaling molecules (e.g., IL-8 and HMGB1) that activate the NF-κB pathway in neighboring cancer cells [137–139]. Notably, NF-κB activation is intricately related to inflammasome signaling and has the potential to mediate the expression of genes associated with pyroptosis [140, 141]. Furthermore, Chalkidi et al. analyzed the transcriptomic profile of CAFs in colitis-associated cancer and revealed the significant enrichment pathways in pyroptosis within CAFs [142]. Thus, the presented evidence indicates that CAFs may possess the potential to regulate pyroptosis indirectly by influencing key molecules within the pyroptotic signaling pathway.

Intriguingly, CD8+ T cells were recently shown to trigger pyroptosis through the release of granzyme [76, 143]. Notably, as the most dominant component of the TME, CAFs have been implicated in modulating the infiltration and behavior of CD8+ T cells. For example, Ou et al. first revealed the spatial and functional characterization of pro-tumorigenic cancer-associated myofibroblasts (myCAFs) in cervical squamous cell carcinoma (CSCC) and discovered that this population was associated with decreased CD4+ and CD8+ T-cell infiltration in CSCC [144]. In pancreatic ductal adenocarcinoma, activated CAFs appear to attract and adhere to CD8+ T cells by secreting CXCL12, preventing their access to cancer cells [145]. Moreover, research indicates that CAFs suppress the activity of CD8+ T cells and can even induce the death of CD8+ T cells through the action of PD-L1/2 and FASL [146, 147]. Therefore, drawing from the aforementioned mechanisms, we hypothesize that CAFs may alleviate the incidence of pyroptosis induced by CD8+ T cells in tumor cells.

Taken together, these findings suggest that CAFs can regulate pyroptosis in tumor cells via indirect signaling pathways or complicated cellular interactions (Fig. 2). However, the specific modulation of cellular pyroptosis induced by CAFs and the intricate molecular mechanisms involved require further investigation.

## Effects of RCD on CAFs

Notably, evidence from numerous studies underscores the fact that dying cells release or expose bioactive molecules on their surface, intricately shaping the dynamics of the TME [148–150]. However, the preceding studies predominantly focused on immune cells, and there remains a paucity of research addressing the impact of cell death specifically on CAFs.

Classically, apoptosis dismantles cells through a non-lytic mechanism and is generally characterized by immunological quiescence [151]. Nevertheless, contemporary findings have proposed that apoptotic cells lead to the generation of apoptotic cell-derived extracellular vesicles (ApoEVs) that can be delivered to adjacent cells, potentially contributing to the modulation of the TME [152]. It has been reported that these vesicles may serve as carriers for the transmission of bioactive molecules and cellular organelles to targeted cells [152, 153]. In the context of gastric cancer, Itoh et al. conducted in vitro co-culture experiments involving CAFs and cancer cells and subsequently isolated ApoEVs from conditioned medium (CM). The findings elucidated that CAFs exhibit a growth-restrictive impact on cancer cells by triggering apoptosis, and intriguingly, ApoEVs from this interaction stimulate the invasive properties of CAFs and cause CAF-led cancer invasion [98]. However, the authors did not provide a detailed exploration of the specific molecular components within the ApoEVs. In contrast, Kim et al. stated that the administration of CM derived from apoptotic lung cancer cells has the potential to attenuate CAF activation and invasion via the inhibition of TGF-β1 signaling pathways as well as the downregulation of MMP-2 and MMP-12 expression [154]. Furthermore, upon interaction with neighboring apoptotic cancer cells expressing Dll1, the activation of Notch1-WISP-1 signaling is initiated in CAFs, thereby exerting anti-invasive effects on lung cancer cells [154]. Hence, the intricate interplay between apoptotic cells and CAFs is characterized by a multifaceted “friend” or “foe” relationship and needs to be carefully differentiated when developing effective strategies for cancer treatment.

Unlike apoptotic cell death, lytic forms of cell death, such as pyroptosis and ferroptosis, lead to membrane permeabilization and cell lysis and the subsequent release of intracellular components and inflammatory cytokines [155, 156]. In the process of cell death, some endogenous molecules known as DAMPs are either released (such as ATP and HMGB1) or exposed on the cell surface (such as calreticulin (CRT) and heat shock protein 90 (HSP90)) [157]. Recent evidence suggests that when exposed to DAMPs, such as ATP, monosodium urate (MSU), H2O2, and necrotic fluid, breast fibroblasts function as DAMP sensors and undergo pro-inflammatory signaling pathway activation via NLRP3 activation, which is particularly intensified by necrotic fluid derived from advanced tumors [130]. Notably, this inflammatory cascade further upregulates αSMA and enhances the activation of fibroblast cells [130, 158]. Moreover, extracellular HMGB1 is the most extensively studied DAMP and acts as a multifunctional protein involved in a variety of physiological and pathological processes [159, 160]. Recently, Chen et al. presented evidence indicating that HMGB1 secreted from breast cancer cells could induce fibroblast activation via its receptor, the receptor for advanced glycation end-products (RAGE), leading to the upregulation of aerobic glycolysis [161]. Additionally, Ren et al. conducted an in vitro cultivation experiment with recombinant HMGB1 and showed an increase in autophagic stimulation in CAFs [139].

Inflammatory cytokines, such as IL-1β and IL-18, which are released during pyroptosis, are also key players in tissue inflammation, tumor immunity, and cancer progression [162–164]. In addition, several studies have investigated the activity of inflammatory cytokines in CAFs. Schauer et al. presented findings indicating that a high level of IL-1β in ovarian cancer stimulates IL-1R1 on adjacent CAFs, leading to the inhibition of P53 and transactivation of the NF-κB signaling pathway in CAFs [165]. As a result, the activated NF-κB signaling pathway further promotes the transcription and release of a diverse array of immunomodulatory chemokines in CAFs [165]. Specifically, IL-1β exhibits the capacity to promote the inflammatory CAFs (iCAF) phenotype, as evidenced by the increased expression of PDPN, PDGFRα, and FAP proteins in CAFs, but did not impact the myCAFs marker protein αSMA [166]. Thus, IL-1β has the potential to activate inflammatory signaling pathways within CAFs and initiate CAF reprogramming.

In addition, Jiang et al. presented that ANO1 could inhibit gastrointestinal cancer ferroptosis in a PI3K-Akt signaling-dependent manner and stimulate the production and secretion of TGF‐β by cancer cells, which in turn recruits CAFs in the TME and confers immunotherapeutic resistance [167]. Interestingly, the suppressed expression and secretion of TGF-β resulting from ANO1 knockdown were reversed by the ferroptosis inhibitor Fer-1, highlighting a potential opposite relationship between ferroptosis and TGF-β release within cancer cells [167]. Moreover, a number of studies have demonstrated the participation of the TGF-β signaling pathway in the formation, activation, migration, and metabolism of CAFs [15, 168, 169]. Hence, ferroptosis may exert regulatory effects on CAFs by releasing TGF-β from cancer cells.

Recently, Mukhopadhyay et al. co-cultured autophagy-inhibited PDAC cells with CAFs and performed a secretome analysis on the supernatant of the autophagy-inhibited PDAC culture medium [120]. The results revealed that autophagy-inhibited PDAC cells could upregulate the level of FPN in adjacent CAFs through the secretion of IL-6, resulting in an increase in the efflux of iron from CAFs and leading to the compensation of the LIP in the autophagy-inhibited PDAC cells [120]. This research introduces, for the first time, the autophagic activity of tumor cells in relation to the iron metabolism of CAFs. Nevertheless, how autophagy-inhibited tumor cells facilitate the secretion of IL-6 and the source of iron in CAFs remains to be further investigated.

**Fig. 3:**
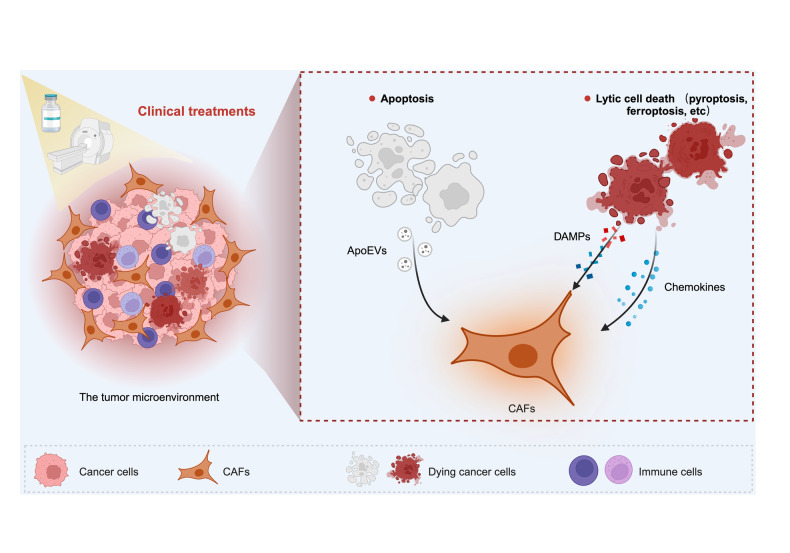
Role of RCD in mediating CAFs. Chemotherapy, radiotherapy, and other clinical chemicals or medications can induce different types of RCD in cancer cells, including apoptosis and lytic cell death. Dying cells release several bioactive molecules, intricately shaping the dynamics and functions of CAFs.

Taken together, RCD plays a significant and influential role in modulating CAFs (Fig. 3), including dynamic alterations in the activation, behavior, function, characteristics, and metabolism of CAFs. Understanding the intricate interplay between RCD and CAF modulation is crucial for unraveling the complexities of the TME and may offer new avenues for cancer treatment.

## Therapeutic implications

Currently, considerable progress has already been made in the field of CAF targeting, and this topic has been reviewed previously [8, 170, 171]. There is no doubt that the exploration of novel therapeutic interventions targeting CAFs holds promise but also presents notable challenges. An important reason is the noteworthy heterogeneity inherent in CAFs, involving both surface marker heterogeneity and functional heterogeneity (Fig. 1). Due to the lack of specific biomarkers in CAFs, an attractive strategy is to switch CAFs to more quiescent fibroblast states through interventions such as all-trans retinoic acid (ATRA) and vitamin D, which have been demonstrated to be effective in restraining CAF functions [172, 173]. Moreover, in preclinical and clinical studies, several drugs have been identified to modulate RCD, thus exerting anti-tumor effects [25, 26, 174]. For example, published results have demonstrated the positive effects of CQ/HCQ in cancer treatment through the suppression of autophagy [175, 176]. Intriguingly, autophagy is also actively involved in the activation and function of CAFs [177], and CAF-regulated autophagy plays a pivotal role in tumor initiation and progression. This opens the way for the development of novel therapeutic approaches that target CAFs and tumor cells simultaneously by regulating the autophagy signaling pathway.

In clinical practice, tumor cells experience several and sometimes intersecting forms of RCD when treated with drugs, and the exact effect of dying cells on the TME remains relatively uncertain. As mentioned above, some endogenous molecules, such as DAMPs and inflammatory cytokines are released during cell death. However, the above molecules may further activate CAFs, thereby causing tumor cells to become resistant to the original treatment. In addition, when tumor cells are suppressed through autophagy, they can also release IL-6, which further contributes to the pro-tumor effects of CAFs [120]. Therefore, in the treatment of tumors in the future, it is crucial to consider the interfering role played by CAFs, and combining the traditional anti-tumor agents with CAF-targeting therapy may improve treatment outcomes.

## Conclusions, challenges and perspectives

Currently, studies have increasingly focused on the importance of the TME for the initiation, progression, metastasis, and drug resistance of cancer. As a prominent component of the TME, CAFs intricately engage in direct and paracrine crosstalk with neighboring cells, manifesting a multifaceted and dynamic influence within the TME. Moreover, extensive investigations have demonstrated the regulatory effect of CAFs on RCD in neighboring cancer cells. Our review summarizes the current knowledge on the interplay between CAFs and four types of RCD. As stated above, CAFs may function as crucial regulators of the RCD process within tumors, whereas dying cells may also confer effects on CAFs, thereby developing a feedback system.

Nevertheless, compared with the issues that have been resolved, there are also multiple challenges that still need to be addressed. First, CAFs exert both pro-tumor and anti-tumor effects on tumor cells by regulating RCD. This indicates the considerable plasticity of CAFs in terms of their function and presents additional difficulties in the search for ways to target CAFs for effective tumor treatment. Second, CAFs can modulate various forms of RCD. Therefore, the variety and complexity of CAF-mediated RCD types within the same tumor microenvironment and the potential crosstalk between these distinct RCD pathways need further investigation. Furthermore, modern techniques, such as single-cell analysis techniques, offer potent means to decipher the heterogeneity of CAFs, which raises the question of whether diverse subpopulations of CAFs elicit similar forms of RCD. Third, in clinical practice, it is noteworthy that anticancer drugs exhibit the capability to induce diverse modes of RCD. For example, anthracycline chemotherapeutic agents have demonstrated the potential to trigger autophagy, apoptosis, and pyroptosis in breast cancer cells [75, 178, 179], and understanding how different RCD modes influence CAF behavior in tumors remains an area that requires further exploration. Last but not least, a substantial portion of research conducted in vitro experiments to investigate the properties of CAFs, which may limit the ability to accurately capture the multifaceted and dynamic roles of CAFs. Notably, when CAFs are isolated from the ECM, the preservation of their biological characteristics becomes a subject of consideration. Moreover, the effect of in vitro culture media on CAFs is still unclear, and the two-dimensional culture model falls short of mimicking the complex cellular interactions that occur in vivo.

Overall, this review provides a brief introduction to CAFs and the core molecular mechanisms involved in four types of RCD. Furthermore, we provide a comprehensive overview of different forms of RCD in tumors that are mediated by CAFs, as well as the effects of these modes of RCD on CAFs, which might offer novel therapeutic avenues for future cancer treatments.
